# Postoperative ileocolic intussusception 48 hours after congenital heart surgery in an infant: a case report

**DOI:** 10.3389/fcvm.2025.1679567

**Published:** 2025-11-12

**Authors:** Huan Li, Mengyu Ke, Jun Yang

**Affiliations:** 1 Department of General Surgery, Wuhan Children’s Hospital (Wuhan Maternal and Child Healthcare Hospital), Tongji Medical College, Huazhong University of Science and Technology, Wuhan, China; 2 School of Medicine, Jianghan University, Wuhan, China

**Keywords:** postoperative intussusception, congenital heart disease, infant, ileocolic, ultrasound, laparotomy

## Abstract

**Background:**

Postoperative intussusception (PI) is a rare but potentially serious complication following congenital heart surgery in infants, often misdiagnosed due to its atypical presentation.

**Case presentation:**

A 3-month-old male infant underwent aortoplasty, ventricular septal defect (VSD) repair, and patent ductus arteriosus (PDA) ligation for congenital heart disease. Approximately 72 h postoperatively, he developed bloody, jam-like stools. Emergency abdominal ultrasound, the first-line imaging modality, revealed ileocolic intussusception. Three attempts at ultrasound-guided hydrostatic saline enema reduction (pressure: 80 cm H_2_O) were unsuccessful, necessitating surgical exploration. Laparotomy identified an 8 cm ileocolic intussusceptum and a 10 cm ischemic ileal segment without a pathological lead point. The necrotic bowel was resected, and an end-to-end anastomosis was performed. The patient recovered uneventfully, was discharged three weeks postoperatively, and demonstrated normal growth at 6-month follow-up.

**Conclusion:**

Prompt identification of postoperative intussusception—a rare complication following non-abdominal surgeries like congenital heart disease repair in infants—is of critical importance.

## 1 Introduction

Postoperative intussusception (PI) is an uncommon complication in children, accounting for approximately 2% of all pediatric intussusception cases (1). Most PI cases are associated with previous abdominal surgery, although isolated occurrences have been reported after thoracic (including cardiac) or cervical procedures (1, 2). Intussusception itself is the most frequent cause of acute intestinal obstruction in infants, with an incidence of 0.3–0.7 per 1,000 live births. Approximately 90%–95% of pediatric intussusception cases are idiopathic, while only 5%–10% are related to identifiable pathological lead points such as polyps or Meckel's diverticulum (3). PI following cardiac surgery is particularly rare. Its pathogenesis is believed to be multifactorial, involving transient intestinal dysmotility induced by anesthetics or opioids, mesenteric hypoperfusion during cardiopulmonary bypass, postoperative inflammation, and electrolyte imbalance (2, 4). Unlike primary intussusception, PI often lacks the classic triad of abdominal pain, vomiting, and bloody stool. Instead, it usually presents with nonspecific signs of progressive intestinal obstruction, which may easily be mistaken for adhesive obstruction (1). This case report, prepared in accordance with the CARE checklist, highlights the diagnostic and therapeutic challenges of postoperative ileocolic intussusception occurring shortly after congenital heart surgery in an infant.

## 2 Case presentation

### 2.1 Patient information

A 3-month-old male infant (weight: 5.1 kg; height: 58 cm) was admitted to the Cardiac Surgery Department of Wuhan Children's Hospital in April 2020. He was born at term via spontaneous vaginal delivery with an uneventful perinatal history (Apgar scores: 9 at 1 min, 10 at 5 min). Cyanosis of the lips had been noted since birth and worsened during crying. Preoperative anthropometric measurements were within normal limits for age.

### 2.2 Clinical timeline

Refer to Table 1.

**Table 1:** Clinical timeline of the children patients.

Time point	Clinical events
Day 0	Congenital heart surgery (aortoplasty, VSD/ASD repair, PDA ligation). Stable postoperative course.
Day 2 (∼48 h)	Extubated after 36 h; feeding initiated at 48 h; mild abdominal distension observed.
Day 3 (∼72 h)	Passage of bloody stool; ultrasound confirmed ileocolic intussusception; hydrostatic reduction failed.
Day 3	Emergency laparotomy performed; resection of 10 cm ischemic ileum; end-to-end anastomosis completed.
Day 10	Tolerated full enteral feeds; normal stool pattern restored.
Post-op 3 weeks	Discharged in good condition.
6-month follow-up	Normal growth and development; no recurrence.
4-year follow-up	Disease stability confirmed at the most recent outpatient visit.

### 2.3 Diagnostic assessment and therapeutic interventions

**Figure 1:**
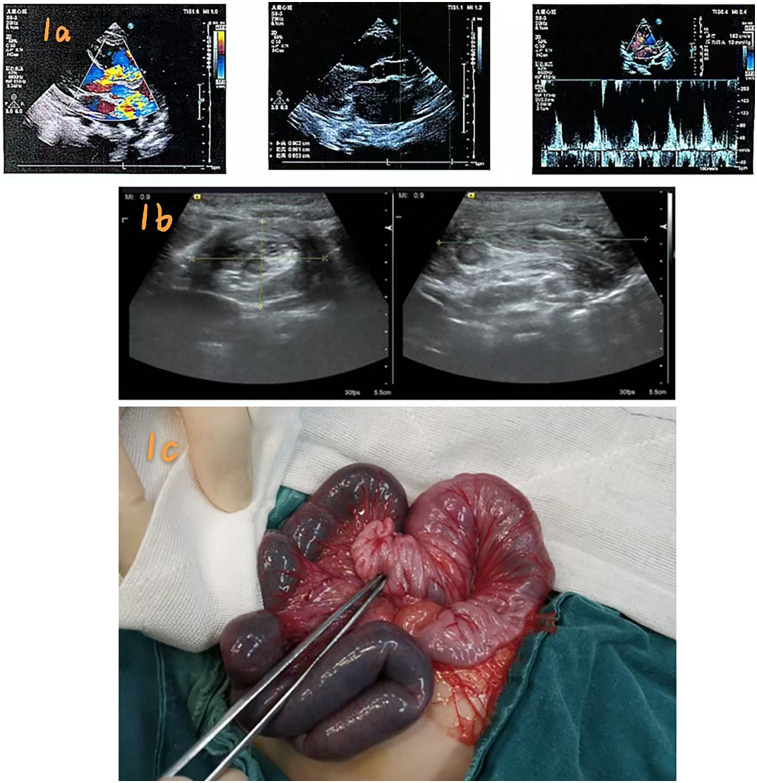
**(a)** Preoperative echocardiography findings; **(b)** Ultrasound “target sign” confirming ileocolic intussusception; **(c)** Intraoperative view showing ischemic ileum and intussuscepted segment.

Preoperative echocardiography revealed aortic coarctation, dilated pulmonary artery, a 10.4 mm VSD, a 5 mm ASD, mild tricuspid regurgitation, mild-to-moderate pulmonary valve regurgitation, mild-to-moderate pulmonary hypertension (mean pulmonary artery pressure: 35 mmHg), and a normal left ventricular ejection fraction (65%) (Figure 1A). The patient's hemodynamic condition was stable, with oxygen saturation of 95%–98% in room air and no clinical evidence of heart failure (e.g., hepatomegaly or peripheral edema). Following exclusion of surgical contraindications, the patient underwent aortoplasty, VSD repair with a tissue patch, ASD repair, and PDA ligation. General anesthesia was maintained with sevoflurane, fentanyl, propofol, and oxycodone. Cardiopulmonary bypass (CPB) was established for 93 min with an aortic cross-clamp time of 76 min. Myocardial protection was achieved with cold crystalloid cardioplegia. Analgesia was provided with low-dose fentanyl (1 µg/kg/h for 48 h). The patient was extubated after 36 h, and enteral feeding commenced 48 h postoperatively. At approximately 72 h postoperatively, physical examination revealed abdominal distension and mild tenderness in the right lower quadrant without guarding or rebound tenderness. Laboratory results showed elevated CRP (35 mg/L; pre-op 10 mg/L), normal white blood cell count (12.5 × 10⁹/L), and normal electrolytes (K^+^ 3.5 mmol/L; Na^+^ 138 mmol/L). Emergency abdominal ultrasound (7.5 MHz transducer) demonstrated the characteristic “target sign” and “pseudokidney sign” in the right lower quadrant, confirming ileocolic intussusception (Figure 1B).

Differential diagnoses included: (1) adhesive obstruction—excluded due to the absence of previous abdominal surgery and the typical ultrasound “target sign”; (2) necrotizing enterocolitis—ruled out given normal bowel sounds, no fever, and the absence of pneumatosis intestinalis; (3) malrotation/volvulus—excluded based on normal superior mesenteric vein orientation on ultrasound. Ultrasound-guided hydrostatic reduction was attempted using warm saline (37 °C) infused through a Foley catheter under a controlled pressure of 80 cm H_2_O. Three 10 min attempts were made, but the “target sign” persisted, indicating reduction failure. Due to ongoing symptoms and the risk of bowel ischemia, urgent laparotomy was performed.

Intraoperatively, a transverse abdominal incision revealed an 8 cm ileocolic intussusception with approximately 10 cm of proximal ileum showing dark purple discoloration consistent with ischemic necrosis. Exploration of the entire intestine revealed no Meckel's diverticulum, polyps, or hypertrophied Peyer's patches (Figure 1C). The ischemic segment was resected, and primary end-to-end anastomosis was performed using 5–0 absorbable sutures. Histopathological examination showed transmural ischemic necrosis, mucosal hemorrhage, and submucosal edema, with no evidence of a pathological lead point. The postoperative course was uneventful. The patient tolerated full enteral feeds by day 10 and was discharged three weeks postoperatively. At 6-month and 4-year follow-up visits, he remained asymptomatic, with normal growth and no recurrence.

## 3 Discussion

The pathogenesis of PI following cardiac surgery is multifactorial. In this case, three mechanisms likely contributed: (1) opioid-induced hypoperistalsis due to continuous fentanyl infusion (2); (2) mesenteric hypoperfusion related to prolonged cardiopulmonary bypass and aortic cross-clamp (4); (3) early enteral feeding that may have triggered premature intestinal motility (1). Age is a key determinant in pediatric intussusception. Idiopathic cases predominate in infants younger than 1 year, while pathological lead points are more common in older children. In contrast to primary intussusception, PI often presents with nonspecific symptoms and lacks the classic triad, increasing the risk of misdiagnosis (1, 2). Ultrasound is the diagnostic modality of choice, offering high sensitivity and specificity without radiation exposure. Computed tomography should be reserved for equivocal cases or when bowel necrosis is suspected (2, 4). A review of 15 pediatric PI cases following cardiac surgery (2018–2025) showed that early diagnosis and timely surgery are key to favorable outcomes, while delays beyond 72 h significantly increase the risk of bowel necrosis (2).

## Data Availability

The datasets presented in this study can be found in online repositories. The names of the repository/repositories and accession number(s) can be found in the article/Supplementary Material.
